# Case Report: Lung Disease in World Trade Center Responders Exposed to Dust and Smoke: Carbon Nanotubes Found in the Lungs of World Trade Center Patients and Dust Samples

**DOI:** 10.1289/ehp.0901159

**Published:** 2009-12-04

**Authors:** Maoxin Wu, Ronald E. Gordon, Robin Herbert, Maria Padilla, Jacqueline Moline, David Mendelson, Virginia Litle, William D. Travis, Joan Gil

**Affiliations:** 1 Department of Pathology; 2 Department of Community and Preventive Medicine; 3 Division of Pulmonary and Sleep Medicine; 4 Department of Radiology and; 5 Department of Thoracic Surgery, Mount Sinai School of Medicine, New York, New York, USA; 6 Department of Pathology, Memorial Sloan-Kettering Cancer Center, New York, New York, USA

**Keywords:** bronchiolitis, carbon nanotubes, interstitial lung disease, small airway disease, WTC

## Abstract

**Context:**

After the collapse of the World Trade Center (WTC) on 11 September 2001, a dense cloud of dust containing high levels of airborne pollutants covered Manhattan and parts of Brooklyn, New York. Between 60,000 and 70,000 responders were exposed. Many reported adverse health effects.

**Case presentation:**

In this report we describe clinical, pathologic, and mineralogic findings in seven previously healthy responders who were exposed to WTC dust on either 11 September or 12 September 2001, who developed severe respiratory impairment or unexplained radiologic findings and underwent video-assisted thoracoscopic surgical lung biopsy procedures at Mount Sinai Medical Center. WTC dust samples were also examined. We found that three of the seven responders had severe or moderate restrictive disease clinically. Histopathology showed interstitial lung disease consistent with small airways disease, bronchiolocentric parenchymal disease, and nonnecrotizing granulomatous condition. Tissue mineralogic analyses showed variable amounts of sheets of aluminum and magnesium silicates, chrysotile asbestos, calcium phosphate, and calcium sulfate. Small shards of glass containing mostly silica and magnesium were also found. Carbon nanotubes (CNT) of various sizes and lengths were noted. CNT were also identified in four of seven WTC dust samples.

**Discussion:**

These findings confirm the previously reported association between WTC dust exposure and bronchiolar and interstitial lung disease. Long-term monitoring of responders will be needed to elucidate the full extent of this problem. The finding of CNT in both WTC dust and lung tissues is unexpected and requires further study.

On 11 September 2001 (9/11), lower Manhattan and parts of Brooklyn were engulfed by a dense cloud of toxic and irritant dust and smoke generated by the collapse of the World Trade Center (WTC) towers (Landrigan et al. 2004; Levin et al. 2002; Lioy et al. 2002). This cloud comprised a complex mix of pollutants, among them the products of combustion of 91,000 L jet fuel, pulverized building materials, cement dust, asbestos, microscopic shards of glass, silica, heavy metals, and numerous organic compounds [see Supplemental Material, Table 1 (doi:10.1289/ehp.0901159)] (Edelman et al. 2003; McGee et al. 2003; Prezant et al. 2002; Reibman et al. 2005).

Adverse health effects have developed since 9/11 in workers and volunteers involved in the rescue, relief, and cleanup at the WTC site and at the Staten Island landfill (the major wreckage depository) (Edelman et al. 2003; Herbert et al. 2006; Landrigan et al. 2004; Lioy et al. 2002; Prezant et al. 2002). The health effects most commonly observed involved the upper and lower respiratory tract. Signs, symptoms, and findings include persistent cough, breathlessness, wheezing, asthma, sinusitis, laryngitis, and irritant-induced asthma, also named reactive airways dysfunction syndrome (RADS) (Herbert et al. 2006; Levin et al. 2002; Prezant et al. 2002). Cases of interstitial lung disease have also been reported, including acute eosinophilic pneumonia, granulomatous pneumonitis, sarcoidosis, and bronchiolitis obliterans (Izbicki et al. 2007; Mann et al. 2005; Rom et al. 2002; Safirstein et al. 2003).

The Mount Sinai WTC Medical Monitoring and Treatment Program (MMTP) was established to provide standardized screening and facilitate treatment of eligible responders who worked or volunteered at the WTC site. There is no systemic or comprehensive roster of all responders similar to the existing records of responders from the New York City uniformed services, such as the Fire Department of New York (FDNY) or New York Police Department, which frequently include their previous health condition. Estimates of the number of responders given by different sources range from 50,000 to 90,000 in total; we believe that the total, including FDNY workers, is likely to have been between 60,000 and 70,000 (Moline et al. 2009). In this article, we report on a case series of seven WTC responders enrolled in the Mount Sinai WTC MMTP who underwent video-assisted thoracoscopic (VATS) procedures at the Mount Sinai Medical Center and whose WTC exposures began on either 11 September or 12 September 2001. As of 11 September 2007, a total of 12,891 responders claiming first- and/or second-day exposure to the WTC pile had monitoring examinations at the Mount Sinai MMTP on or before 11 September 2007. Of these responders, one underwent VATS with biopsy in 2005, and six underwent VATS procedures between 1 January and 31 October 2007, because of severe pulmonary symptoms, impairment, or unexplained radiologic findings. We describe here the histopathologic patterns associated with these severe forms of respiratory impairment.

As part of our overall biopsy examination, we performed mineralogic analyses of the tissue from seven individuals believed to have been previously healthy who developed signs of respiratory impairment after sustaining WTC exposures. Additionally, we obtained and analyzed dust specimens collected on the site (DS) and examined old specimens (controls for old cases; COC) unrelated to the WTC disaster that were routinely submitted to our laboratory for asbestos burden analysis (*n* = 40) or obtained for research purposes from autopsy or surgical specimens (*n* = 20) of patients without history of WTC exposure.

## Material and Methods

### Patients

All seven patients included in this study had either never smoked or had smoked only in the remote past. They were exposed to WTC dust/smoke beginning on either 11 September or 12 September 2001, and were sufficiently ill to undergo VATS biopsy. The decision to pursue a VATS biopsy was made by their attending pulmonologist, based on poor respiratory function with high-resolution computerized tomography (HRCT) changes suggestive of interstitial disease, marked worsening of the patient’s condition, or any nodular radiologic patterns. All patients signed release forms authorizing research studies and were again contacted to confirm their willingness to participate. The patients continue to be followed. Permission for publication was obtained from the Mount Sinai Medical School Institutional Review Board (07-0788 0001 01 PA X).

### Clinico-pathologic workup

The following information was obtained from the medical records: age, sex, occupation, smoking history, comorbidities, WTC exposure (starting date and length of time of exposure, whether caught in the original dust cloud from the WTC building collapse on 11 September), chief complaints at presentation, pulmonary function tests (PFTs) including forced expiratory volume in 1 sec (FEV_1_) and forced vital capacity (FVC), diffusing capacity of the lung for carbon monoxide (DL_CO_), and HRCT findings before biopsy and serologies for autoimmune disease.

After it had been determined that a VATS biopsy was needed, wedges of lung from the right upper, middle, and lower lobes were obtained and submitted to the pathology service of Mount Sinai Hospital. These specimens were processed for light microscopy, following the routine procedures of surgical pathology specimens stained with hematoxylin and eosin, and diagnosed by pathologists from the hospital service. Later, all three anatomic pathologists in this study (M.W., J.G., and W.T.) jointly reviewed all biopsies.

### Electron microscopy mineralogic analysis

A representative block of paraffin-embedded tissue was taken from each case and digested for mineralogic and asbestos fiber burden analysis. The paraffin was removed from the tissue and the hydrated lung tissue was blotted and weighed. The tissue was then submerged in potassium hydroxide for digestion. The digested tissue was centrifuged to separate the nonsolubilized materials from reagents and solubilized materials. The precipitate was washed five times with distilled water. The digested lung material was resuspended in 10 mL distilled water, and 10-μL samples were removed from each and placed on formvar-coated nickel grids. The grids were analyzed by transmission electron microscopy using a standard fiber-counting protocol. Positive and negative control samples were prepared from the same distilled water used to wash the sample. Verification techniques of fiber counting were used for quality control and quality assurance. A total of 400 grid openings were scanned for each lung specimen on at least four separate grids at magnifications of either 10,000 or 20,000. The fibers, if present, were counted. All other structures were identified morphologically and by energy-dispersive spectroscopy (EDS) and counted. Chrysotile asbestos was confirmed by EDS and selected area electron diffraction. Identification of carbon nanotubes (CNT) was based on comparison with a positive control sample provided by A. Shvedova from the National Institute for Occupational Safety and Health (NIOSH) and on the images and descriptions found in two publications (Lam et al. 2006; Poland et al. 2008). Seven DS collected at different sites of the WTC (generously provided by R. Nolan, Environmental Sciences, City University of New York) were also observed by analytic electron microscopy for the presence of structures, particularly CNT. In addition, we retrospectively looked at 40 COC—recent, previously analyzed cases of construction workers (carpenters, electricians, laborers, plumbers) believed to have been exposed to the inhalation of asbestos fibers, evaluated for asbestos burden, and found to be positive for that substance. To assess background levels in persons with no history of ever being exposed to asbestos or WTC dust, we also reevaluated 20 lung tissue samples from either lung resections or autopsies used to study for research purposes. Therefore, the total of reevaluated old cases was 60.

## Results

### Case reports

A summary of patient clinical, WTC exposure, PFT findings and radiologic findings on HCRT imaging is presented in Supplemental Material, Table 2 (doi:10.1289/ehp.0901159). Table 1 summarizes the pathologic/mineralogic/asbestosis fiber burden analysis. All patients affirmed that their symptoms had developed after the 9/11 attack and denied history of asthma or of any other significant pulmonary conditions except as shown for Patient B.

#### Patient A

Patient A is a 59-year-old female nurse who was caught in the dust cloud for 4 hr on 9/11. She also lives three blocks from the WTC site. She worked on the pile or in the pit at the WTC site for 41 days, or 352 hr, giving first aid to workers and pedestrians. The patient presented to the WTC MMTP in November 2006 with shortness of breath, hoarseness, cough, wheezing, and chest tightness. After 11 September, she developed asthma, bronchitis, and pneumonia. Her past medical history is significant only for hypothyroidism. The patient is a nonsmoker.

**Figure 1:**
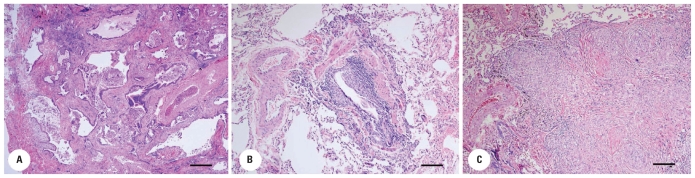
Light photomicrographs of representative histopathology of patient lung tissue of the three groups. Bar = 10 μm. (*A*) Honeycomb fibrosis, Patient A. There is extensive dense fibrosis with cystic remodeling of the lung parenchyma. The cysts are lined by pseudostratified respiratory epithelium, and are either empty or filled with mucous and/or inflammatory cells. A bronchiole is seen in the center near the artery. (*B*) Chronic bronchiolitis, Patient D. There is marked chronic inflammation in the submucosa with interstitial clusters of macrophages and metaplasia of the respiratory epithelium. (*C*) Granuloma, Patient G. Poorly formed nonnecrotizing granulomas in interstitium.

PFTs (8 August 2007) were consistent with severe restrictive lung disease, with FVC 46%, FEV_1_ 51%, and DL_CO_ 38%. HRCT revealed interstitial fibrosis with a subpleural distribution with prominent mediastinal nodes. Lung biopsy showed honeycombing, severe peripheral fibrosis, and peribronchiolar usual interstitial pneumonitis (UIP)-like fibrosis (Figure 1B; Supplemental Material, Figure 1B (doi:10.1289/ehp.0901159). Mineralogic analysis revealed silicates 165,600/g and CNT 110,400/g.

The patient was last seen on 9 September 2009 and continued to have severe dyspnea on effort, aggravated by early satiety and abdominal fullness. PFT showed worsening in her lung function, with FVC 40%, FEV_1_ 46%, and DL_CO_ 29%. CT scan of chest revealed progression of interstitial fibrosis and decreased lung volumes. The patient has been referred for lung transplant, but active lung transplant listing has been postponed because of her subjective well-being and physical appearance despite progression of disease.

#### Patient B

This patient is a 59-year-old male who worked as an electrician. He arrived at the WTC site on day 1 and worked for 90 days, or 1,080 hr, adjacent to the pile/pit. He worked as an engineer at the site repairing and accessing damage to signals, generators, and power cables for New York City transit. He presented to MMTP in March 2006 with complaints of dyspnea, cough, throat irritation, and wheezing. He has a past medical history of pneumothorax, “cyst in the lung,” and arrhythmia. After 9/11, he developed sleep apnea (on continuous positive airway pressure). The patient is a nonsmoker.

PFTs are consistent with a mild restrictive and obstructive pattern, along with oxygen desaturation with exercise (97%–90%). His chest radiograph revealed nodules. HRCT (6 November 2006) revealed multiple small nodules (seven), prominent interstitial markings, and bronchiectasis. Lung biopsy showed interstitial fibrosis, mostly bronchiolocentric, with multiple patterns consistent with UIP, nonspecific interstitial pneumonitis (NSIP) [Supplemental Material, Figure 1F (doi:10.1289/ehp.0901159)], and hypersensitivity pneumonitis. Mineralogic analysis revealed CNT 3,450/g, silicates 165,600/g, and no asbestos fibers

At a follow-up visit in 18 September 2009, the patient complained of shortness of breath, which remained unchanged, and worsening joint pain. His PFTs were FVC 86%, FEV_1_ 80%, and DL_CO_ 82%. The patient was referred to a rheumatologist, and standard tests for autoimmunity were negative. Oxygen desaturation is still present but has improved: 98%–93%. CT scan of the chest shows mild worsening of the interstitial markings. Bronchiectasis and nodules are unchanged.

#### Patient C

Patient C is a 49-year-old male who worked in building trades, who presented in February 2007 with complaints of cough since 2005 and dyspnea on exertion. He worked adjacent to the pile 10–12 hr/day for 8 days after 9/11 (102 hr) as a custodian at the WTC site, covering up vents and sweeping out dust. He has a past medical history of myocardial infarction. He is a nonsmoker.

PFTs (23 August 2007) were consistent with severe restrictive lung disease, with FVC 49%, FEV_1_ 50%, and DL_CO_ 34%. HRCT reveals subpleural interstitial linear and ground glass changes. Enlarged lymph nodes were also present. Lung biopsy showed peribronchiolar fibrosis [Supplemental Material, Figure 1D (doi:10.1289/ehp.0901159)], NSIP-type, with extensive lymphocytic infiltrates and bronchiolitis. Mineralogic analysis revealed chrysotile asbestos 36,800/g; silicates 184,000/g, and CNT 230,000/g.

This patient was last seen 2 March 2009 and continues to have limited functional capacity, with continued dyspnea on exertion (DOE) and overall disease progression. He is on immunosuppressive medication. PFTs show FVC 41%, FEV_1_ 45%, and DL_CO_ 34%. HRCT shows progression of parenchymal disease.

#### Patient D

This patient is a 40-year-old male health care worker who was caught in the dust cloud of the WTC collapse. He worked on the WTC site from 11 September 2001 until February 2002, for a total of 1,080 hr. He worked on the pile/pit and the office of chief medical examiner (OCME), digging on the pile and identifying remains in the morgue. He presented in May 2006 with 3 years of DOE, dry cough, sore throat, hoarseness, wheezing, worsening gastroesophageal reflux (GERD), and diarrhea. After 11 September 2001, the patient developed sleep apnea, chronic bronchitis, vocal cord dysfunction, posttraumatic stress disorder, chest pain, difficulty breathing, heartburn, unstable angina, GERD, shortness of breath, and cough. The patient is a nonsmoker.

**Figure 2:**
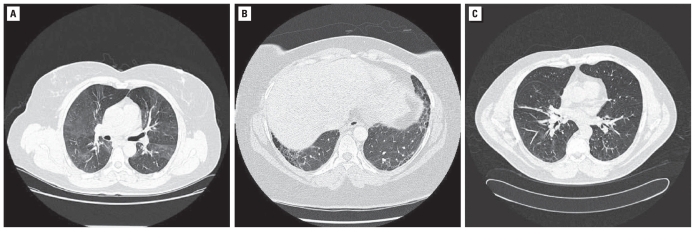
Representative HRCT images. (*A*) Air trapping (mosaic pattern) in Patient D (end expiratory). (*B*) UIP-like pattern in Patient E. (*C*) Sarcoid-like pattern in Patient G.

PFTs before VATS biopsy showed mild to moderate restrictive lung disease, with FVC 65% and FEV_1_ 70%; DL_CO_ was not available. HRCT revealed a mosaic lung (air trapping) (Figure 2A). Lung biopsy showed bronchiolitis [Supplemental Material, Figure 1A (doi:10.1289/ehp.0901159)], and mild peribronchiolar fibrosis (Figure 1A). Mineralogic analysis revealed CNT 11,040/g, silicates 27,600/g (plus calcium sulfate in large amounts), and chrysotile asbestos 5,520/g.

Patient D was last seen in follow-up in July 2008 and continued to complain of significant DOE. Cardiopulmonary exercise testing, PFTs, and oxygen saturation were normal.

#### Patient E

The patient is a 46-year-old female law enforcement officer. She was caught in the original cloud and worked on 9/11 for 12 hr. She worked adjacent to the pile/pit and the OCME for a total of 41 days, or 352 hr. The patient stated that her main activities at the WTC site included working at the morgue. The patient presented to the MMTP in October 2004 with complaints of shortness of breath and dyspnea on effort with some chest pain and wheezing. The patient is a nonsmoker.

After 11 September 2001, the patient developed asthma, breathing problems, and dysphagia. PFTs revealed an FVC of 65%, FEV_1_ 69%, and DL_CO_ 46%. HRCT revealed peripheral changes suggestive of UIP [Figure 2B; see also Supplemental Material, Figure 1C (doi:10.1289/ehp.0901159)]. Lymph nodes were mildly enlarged. Mineralogic analysis revealed chrysotile asbestos 110,400/g and 110,400/g silicates. Lung biopsy showed focal areas of fibrosis with fibroblastic foci small granulomas, nonnecrotizing, and peribronchiolar lesions. Airways contained mucopurulent material.

She was last seen by her pulmonologist in June 2009, with stable complaints. Her CT scan findings from November 2008 are stable compared with February 2006, as were her PFTS. Resting oxygen saturation was 98%.

#### Patient F

Patient F is a 55-year-old female custodian and asbestos handler. She worked adjacent to the pile/pit on the WTC site from 12 September to 23 November for 660 hr. She worked as a custodian and in dust suppress at the WTC site. In October 2003, the patient presented with dry cough, wheezing, decreased exercise tolerance, and sleep apnea. Her past medical history is noncontributory. The patient is a former smoker (31 pack-years).

PFTs are consistent with normal spirometry and diffusing capacity. HRCT demonstrated marked mosaic pattern consistent with air trapping, as well as old calcified granuloma. Lung biopsy showed small airways disease, respiratory bronchiolitis [Supplemental Material, Figure 1E (doi:10.1289/ehp.0901159)], peribronchial bronchiolar metaplasia. Lung parenchyma was unremarkable. Mineralogic analysis showed no relevant findings.

Patient F was last seen in June 2009. She continued to have significant upper respiratory complaints, along with increased cough, chest tightness and wheezing, sinusitis, and generalized pain. She is disabled.

#### Patient G

Patient G is a 55-year-old male law enforcement officer who worked on the WTC site on 11 September for 18 hr. The patient worked on and adjacent to the pile/pit, and his main activities included working in bucket brigade and security at the WTC site. The patient initially complained of dyspnea on exertion, cough, wheezing, and chest tightness. The patient is a nonsmoker.

PFTs revealed minimal restriction and a positive bronchodilator response consistent with RADS. HRCT revealed multiple nodes (Figure 2C), some calcified, and peribronchial interstitial disease. Lung biopsy shows nonnecrotizing epithelioid granulomas (Figure 1C) and small airways disease. Mineralogic analysis reveals magnesium silicate 63,086/g and chrysotile asbestos 11,829/g.

This patient was last seen in July 2009 with complaints of worsening cough and shortness of breath. HRCT shows a miliary pattern of innumerable tiny nodular densities in both lungs. Multiple small calcified lymph nodes in mediastinum, fibrous bands, and ground glass areas were also seen. A gallium scan shows faint uptake in hilum but none in parenchyma. He was evaluated for sarcoidosis with negative results. A Kveim test was negative. No subsequent manifestations consistent with sarcoidosis have been noted.

### Electron microscopy mineralogic findings

**Figure 3:**
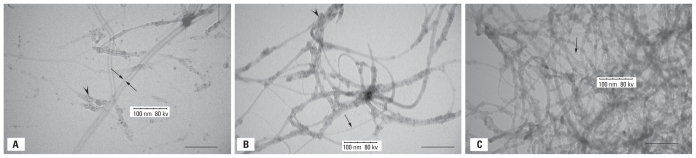
Electron micrographs of CNT. These appear as tangled long transparent hairlike ropes and stacks of single-walled CNT. All three representative graphs were taken at a magnification of 100K. Single CNT in each graph are indicated by thin arrow. The arrowheads depict metal particles. Bar = 100 nm. (*A*) Digested patient lung tissue. (*B*) WTC dust sample. (*C*) Control sample from NIOSH.

A summary of the mineralogic analysis in correlation with pathologic features is given in Table 1. Four of the seven WTC dust samples contained CNT. The lung specimens of three of the patients with interstitial disease (Patients A, B, and C) contained CNT (Figure 3A) virtually identical to those of the dust samples (Figure 3B) and of the positive control sample (Figure 3C). CNT seen were all single-walled and of various lengths. They were mostly ropelike under electron microscopy. The fourth patient with CNT had mild chronic bronchiolitis and occasional peribronchiolar and submucosal fibrosis (Patient D). The nanotubes identified in patient lung samples appear mostly in single or pairs. The single nanotubes are indicated by arrows in all graphs of Figure 3. Most CNT seen in the CNT-positive control and WTC dust samples were stacks of single nanotubes, thus appearing to be variable in thickness. No CNT were found in the lungs of either the 40 construction workers or the 20 negative control samples.

Of the patients with interstitial disease, all had large amounts of aluminum and magnesium silicates in an unusual platy configuration, ranging from 27,600 to 184,000/g wet weight of lung. As a comparison, we reexamined for the presence of CNT in 40 samples taken from unrelated workers from diverse construction trades suspected for asbestos-related disease. These patients were known to have been exposed to asbestos, and most of these 40 patients had a high lung burden of asbestos fibers. Less than 10%, however, had platy aluminum and magnesium silicates similar to those seen in WTC patients. Furthermore when they were seen in the control samples, they were present in small quantities (< 1,000/g wet weight of lung).

## Discussion

In this case report, we describe seven previously healthy WTC responders who developed lung disease after sustaining heavy first- and second-day exposures to WTC dust and debris. We summarize our findings as follows: *a*) CNT were found in some WTC-exposed patients with persistent lung disease with confirmation by positive controls and in the WTC dust. This finding was present in responders with extensive interstitial/parenchymal abnormalities (three of four patients) and in one of two patients with small airway disease, but not in the patient with nonnecrotizing granulomas. *b*) We found a higher amount of aluminum silicate and magnesium silicate in all four patients with diffuse interstitial/parenchymal abnormalities compared with those with small airway disease and granuloma. Chrysotile asbestos fibers were also found in four of seven patients in quantities modestly higher than background levels of asbestos in the New York City population. In interpreting these findings, however, it should be noted that mineralogic analysis of the lungs can be biased because of sampling variability within the lung itself.

The seven biopsies revealed multiple, partly repeating and mixed histologic patterns. *a*) Small airways disease was present in almost all cases at different levels of severity, and we highlight patients D and F, in whom it was the main finding. Radiologically, these two patients presented with a mosaic pattern indicative of air trapping similar to that previously described in 25 WTC responders (Mendelson et al. 2007). Small airways disease is a condition that pathologically can be controversial and includes bronchiolitis (Fenton et al. 2007; Niewoehner et al. 1974; Wright et al. 1992). It may well be the most frequent injury pattern in exposed patients with severe respiratory impairment. *b*) Interstitial disease was present in four cases (Patients A, B, C, and E), characterized by a generally bronchiolocentric pattern of interstitial inflammation and fibrosis of variable severity. The lungs of these patients contained large amounts of silicates, and three of them showed nanotubes. The peripheral alveolar remodeling and fibrosis could variably be characterized as honeycombing, UIP- or NSIP-like, had some fibroblastic foci, or even resembled hypersensitivity pneumonitis (which was ruled out by pertinent serologies in all cases). Patient E even showed small numbers of poorly organized granulomas in one of the lobes and a mediastinal lymph node. The number of interstitial lymphocytes was variable, and some evidence of bronchiolitis was generally present. The bronchiolocentric characteristics seem consistent with an inhalation-related etiology. *c*) We had only one patient with the principal diagnosis of granulomatous disease (Patient G), although Patient E also revealed some small scattered granulomas. In the intervening years, this patient has developed evidence of interstitial disease, in the form of a miliary pattern of minute nodules, but no signs of sarcoidosis, including a negative Kveim test. Mineralogic studies may reflect sampling heterogeneity. Granulomatous disease has been previously described after WTC exposure (Izbicki et al. 2007; Safirstein et al. 2003).

Determination of the epidemiology of interstitial lung diseases and exact incidence rates remains challenging. Although several studies investigating incidence rates in interstitial lung disease have been conducted, uncertainty exists because of their reliance on databases and diagnoses codes (Coultas et al. 1994; Gribbin et al. 2006; Raghu et al. 2006). Difficulties because of changes in disease definitions and histopathologic classifications have further complicated the issue, as well as differences in epidemiologic design of studies, limited large-scale epidemiologic data for specific types of interstitial lung disease, and registration bias (Demedts et al. 2001).

We have not sought to describe the incidence of interstitial disease in the WTC MMTP patients. The sudden onset of six biopsies of our cases in January 2007 and the unexpected end of such cases, in October of the same year, is likely a reflection of the availability of federal funding for comprehensive treatment for WTC responders, which only first became available in the MMTP in November 2006. Patients may have sought care outside of the MMTP in earlier years, and some patients might have been too ill to visit the MMTP. Given these limitations, these cases are not an accurate representation of the time of onset. Whether they are an accurate representation of the total burden in this cohort remains to be seen, but it is unlikely, given the fact that physicians, including pulmonologists, have different criteria with regard to ordering diagnostic tests such as CT scans, diffusion measurements, and VATS biopsies.

Electron microscopic mineralogic analysis revealed CNT in WTC responders with extensive interstitial/parenchymal abnormalities (three of four patients) and in one of two patients with small airway disease, but not in the patient with nonnecrotizing granulomas. Nanomaterials such as CNT have many potential applications in electronics, computer, and aerospace industries because of their desirable electrical, mechanical, and thermal properties (Lam et al. 2004). CNT are hydrophobic carbon cylinders with a diameter of a few to 200 nm and variable length depending on their degree of aggregation (Florito et al. 2006; Li et al. 2007; Mercer et al. 2008; Murr et al. 2005; Shvedova et al. 2008a; Tian et al. 2006). There are single-walled carbon nanotubes (SWCNT), consisting of one such cylinder, and multiwalled carbon nanotubes (MWCNT), composed of cylinders concentrically stacked and in the form of ropes with a common long axis. They can either be commercially synthesized or can develop spontaneously over flames and high temperatures in the presence of carbon and a metal catalyst.

CNT of commercial origin, common now, would not have been present in substantial numbers in the WTC complex before the disaster in 2001. However, the high temperatures generated during the WTC disaster as a result of the combustion of fuel in the presence of carbon and metals would have been sufficient to locally generate large numbers of CNT. This scenario could have caused the generation of CNT that we have noted in the dust samples and in the lung biopsy specimens.

Experimental studies, both *in vitro* and in animals, show the potential toxicity of some types of CNT (Florito et al. 2006; Li et al. 2007; Mercer et al. 2008; Murr et al. 2005; Shvedova et al. 2008a, 2008b; Tian et al. 2006). They are believed to be proinflammatory, fibrosing, and capable of inducing granulomas when inhaled in mouse models (Mercer et al. 2008). Shvedova et al. (2008b) observed that inhalation of SWCNT resulted in a 4-fold increase in fibrosis, along with collagen deposition, in the peribronchial and interstitial areas in the lungs of mice when compared with exposure by aspiration of SWCNT. CNT introduced into the abdominal cavity of mice can cause asbestos-like pathogenicity (Poland et al. 2008). Very recently, the Berkeley group has shown that oxidative stress can be induced by zero-valent iron (Fe) nanoparticles and Fe(II) in human bronchial epithelial cells (Keenan el al. 2009), which may contribute to the chain reaction of oxidative damages.

There is evidence that CNT may contribute to granuloma formation, although this may depend on the type of CNT present. In a study by Lam et al. (2004), granuloma formation and interstitial inflammation was observed in four of nine mice who were administered a high dose (0.5 mg) of CNT compared with mice administered a low dose (0.1 mg) of CNT. Similar findings were also observed in a study conducted by Muller et al. (2005), where granulomas, completely or partially blocking the bronchial lumen, were observed in the bronchi of rats 60 days after intratracheal instillation of MWCNT. Additionally, ground MWCNT, administered to rats via intratracheal instillation, were better dispersed in the parenchyma and also produced granulomas in the interstitial tissue (Muller et al. 2005). Shvedova et al. (2005) showed that epitheloid granulomas, which were rapid, progressive, and dose dependent, were observed at sites where SWCNT aggregates were deposited after pharyngeal aspiration of SWCNT in mice. Furthermore, diffuse interstitial fibrosis with alveolar thickening was also observed in areas distant from the site where SWCNT aggregates were deposited (Shvedova et al. 2005). In most of these studies, carbon black particles used as reference particles did not produce any granulomas (Lam et al. 2004; Muller et al. 2005; Shvedova et al. 2005). However, CNT were not found in Patient G who had granulomas, perhaps because of inadequate sampling of the tissue. Mediastinal granulomas were described in prior studies by other WTC-exposed individuals (Izbicki et al. 2007).

Aluminum and magnesium silicates were present in six of seven patients in higher amounts than normally seen in construction workers. Although these may be related to construction material, some of them were in an unusual platy configuration rarely found in our COC from construction workers. Markedly higher levels (> 100,000/g) of aluminum silicate and magnesium silicate were found in Patients C–F.

The etiology of the conditions described in WTC responders cannot be fully elucidated, because our analysis does not include toxic soluble gases. In this case report we identified the presence of unexpected CNT, silicates, and other elements, but it remains unclear whether any of these compounds may have caused the lung pathology. The combination of compounds may have increased the likelihood that individuals would develop pulmonary impairment. Further surveillance of individuals with these exposures should provide us with additional answers.

The finding of carbon nanotubes in four of seven WTC patients in concentrations ranging from 11,000/g to 230,000/g of wet weight and in four of seven dust samples collected from the WTC site, although unique and important, cannot be construed as evidence for WTC exposure. Likewise, their absence should not be used as evidence of lack of exposure. A retrospective check of specimens in unrelated asbestos cases evaluated over the past 20 years was negative for CNT. Recently, however, small numbers of CNT have been identified in control specimens from the tri-state area (New York, New Jersey, Connecticut) at minimal concentrations (Gordon R, unpublished data). These might originate in the combustion engines of automobiles (Lam et al. 2006).

## Conclusion

The predominant finding of interstitial lung disease on pathology and presence of similar materials in most of the mineralogic samples including CNT and platy silicates suggests that WTC exposure may have contributed to the development of lung disease. Furthermore, there is consistency of the histologic patterns with those seen in inhalation-related injury. The frequency of small airways disease described in epidemiologic studies of WTC responders and the slow and relatively mild progression and long survival of the patients, which now appear to be exceeding survival of some with idiopathic interstitial lung disease, suggest that exposures at the WTC site might have caused these findings. Further follow-up of WTC-exposed individuals is critical to determine the overall health effects related to WTC exposure.

## Figures and Tables

**Table 1:** Summary of findings in pathology and minerologic/asbestos fiber burden analyses.

Patient	Pathologic summary	Mineralogic and asbestos fiber burden analysis
A	UIP-like, HP-like	AS and MS: 165,600/g
		CNT: 110,400/g
B	UIP-like, NSIP-like	AS and MS: 165,600/g
		CNT: 3,450/g
C	NSIP-like (cellular > fibrosing)	CA: 36,800/g
		AS and MS: 184,000/g
		CNT: 230,000/g
D	Mild chronic bronchiolitis; occasional peribronchiolar and submucosal fibrosis	CA: 5,520/g
		AS and MS: 27,600/g
		CNT: 11,040/g
		CS: large amount
E	UIP-like, HP-like	CA: 110,400/g
		AS and MS: 110,400/g
F	Peribronchiolar metaplasia and respiratory bronchiolitis	CA: ND
		AS, CS, CNT: ND
G	Sarcoid-like	CA: 11,829/g
		MS: 63,086/g

Abbreviations: AS, aluminum silicate; CS, calcium sulfate; CA, chrysotile asbestos; HP, hypersensitivity pneumonia; MS, magnesium silicate; ND, none detected; NSIP, nonspecific interstitial pneumonitis; UIP, usual interstitial pneumonitis.
